# Drug Repositioning of the α_1_-Adrenergic Receptor Antagonist Naftopidil: A Potential New Anti-Cancer Drug?

**DOI:** 10.3390/ijms21155339

**Published:** 2020-07-27

**Authors:** Romane Florent, Laurent Poulain, Monique N’Diaye

**Affiliations:** 1 Normandie Univ, UNICAEN, INSERM U1086 ANTICIPE (Interdisciplinary Research Unit for Cancers Prevention and Treatment), BioTICLA axis (Biology and Innovative Therapeutics for Ovarian Cancers), 14000 Caen, France; romane.florent@unicaen.fr (R.F.); l.poulain@baclesse.unicancer.fr (L.P.); 2 UNICANCER, Comprehensive Cancer Center François Baclesse, 14000 Caen, France; 3 Biological Ressources Center «OvaRessources», Comprehensive Cancer Center François Baclesse, 14000 Caen, France

**Keywords:** cancer, drug repurposing, α_1_-adrenergic receptor antagonists, naftopidil

## Abstract

Failure of conventional treatments is often observed in cancer management and this requires the development of alternative therapeutic strategies. However, new drug development is known to be a high-failure process because of the possibility of a lower efficacy than expected for the drug or appearance of non-manageable side effects. Another way to find alternative therapeutic drugs consists in identifying new applications for drugs already approved for a particular disease: a concept named “drug repurposing”. In this context, several studies demonstrated the potential anti-tumour activity exerted by α1-adrenergic receptor antagonists and notably renewed interest for naftopidil as an anti-cancer drug. Naftopidil is used for benign prostatic hyperplasia management in Japan and a retrospective study brought out a reduced incidence of prostate cancer in patients that had been prescribed this drug. Further studies showed that naftopidil exerted anti-proliferative and cytotoxic effects on prostate cancer as well as several other cancer types in vitro, as well as ex vivo and in vivo. Moreover, naftopidil was demonstrated to modulate the expression of Bcl-2 family pro-apoptotic members which could be used to sensitise cancer cells to targeting therapies and to overcome resistance of cancer cells to apoptosis. For most of these anti-cancer effects, the molecular pathway is either not fully deciphered or shown to involve α1-adrenergic receptor-independent pathway, suggesting off target transduction signals. In order to improve its efficacy, naftopidil analogues were designed and shown to be effective in several studies. Thereby, naftopidil appears to display anti-cancer properties on different cancer types and could be considered as a candidate for drug repurposing although its anti-cancerous activities need to be studied more deeply in prospective randomized clinical trials.

## 1. Drug Repurposing: A Promising Tool for Cancer Management

Despite many improvements in prevention and therapy, failure of conventional treatments is often observed and cancer management requires the development of new therapeutic strategies. At this time, development of targeting therapies like PARP (Poly (ADP-ribose) polymerase) inhibitors, anti-angiogenic molecules or immunotherapy are booming [1,2,3]. Nevertheless, new drug development is time-consuming, expensive and is a high failure process because of the possibility of a lower efficacy than expected for the drug (for example if compensatory feedback loops take over) or appearance of non-manageable side effects. These hurdles mean that only one of every 5000–10,000 proposed anti-cancer drugs are approved by the Food and Drug Administration (FDA) [4].

In this context, drug repurposing (thank to computational and experimental approaches) aims at identifying new uses for drugs already approved or investigated for a particular disease and can be a mean to overcome those barriers. Actually, drug repurposing alleviates the pre-clinical steps of a classical drug development allowing significant time savings. Moreover, toxicity and safety profiles of the repurposed molecule are already known, as well as its pharmacokinetic and pharmacodynamic properties, reducing the risk of failure because of deleterious effects [5,6].

A bibliometric analysis conducted to appreciate the use of drug repurposing revealed that around 21,000 of the chemicals related to disease were associated with more than 1 disease suggesting a drug repurposing strategy. Among them, nearly 200 were connected for more than 300 diseases each [7]. This strategy has been successfully exploited many times and one of the most known examples is that of the phosphodiesterase inhibitor sildenafil, commercialized as Viagra by Pfizer and initially used for angina pectoris treatment. This drug has successfully been repurposed for erectile dysfunction management [8].

As for cancer management, recycling of drugs allows a rapid translation into clinical trial and appears as a promising strategy. In fact, some drugs used for non-cancerous diseases are also able to target the hallmarks of cancer cells including proliferation, resistance to cell death, angiogenesis, migration/invasion, tumour promoting inflammation and deregulated cellular metabolism and thereby represent promising candidates [9]. Thereupon, several non-cancer drugs exhibit anti-cancerous activities, such as the antidiabetic metformin which is efficient on various cancer types, notably lung, breast, prostate, colorectal and pancreatic cancers [10]. Actually, metformin seems to reduce cancer cells growth by activating the Adenosine Monophosphate-activated protein kinase (AMPK) and thereby modulating the activity of several metabolic pathways required for tumour growth [11]. Moreover, raloxifene used firstly for osteoporosis in postmenopausal women treatment was approved by the FDA for breast cancer prevention in high risk women [12] and the antiparasitic agent ivermectin or the anti-fungal agent itraconazole are already studied as potential repurposed drugs for ovarian cancer management [13].

In this context, several studies demonstrated the potential anti-tumour activity exerted by α_1_-adrenergic receptor (α_1_-AR) antagonists [14] which could make them to be considered as a potential strategy for cancer management and good candidates for drug repurposing concept.

## 2. Alpha_1_-Adrenergic Receptor Antagonists

The α_1_-AR are divided in three subtypes: α_1A_, α_1B_ and α_1D_ which are expressed in several human tissues. The distribution of these receptors has been performed largely by analysis of mRNA expression and revealed that α_1B_ subtype is highly expressed by spleen, kidney, heart and brain tissues. Smooth muscles express the three α_1_-AR subtypes with a predominant α_1A_ and α_1D_ expression, which are also found in aorta and cerebral cortex tissues [15,16,17].

These receptors are classically coupled to Gq/11 proteins (but are also reported to be coupled to Gi proteins) and they are activated by catecholamines (adrenaline, noradrenaline, dopamine) [17,18]. The ligand binding on the receptor induces the dissociation of the Gq/11 protein which activates the phospholipase C (PLC). This enzyme cleaves its substrate: the membrane phospholipid phosphatydylinositol-4,5-bisphosphate (PIP2) into inositol 1,4,5-triphosphate (IP3) and diacylglycerol (DAG). The IP3 binds to its receptor, the IP3-receptor (IP3-R), located at the endoplasmic reticulum (ER) surface, allowing the exit of calcium from this organelle to the cytosol. As for DAG, it stimulates Protein Kinase C (PKC) that phosphorylates in its turn several proteins, leading to a cell specific response. DAG can also activate various calcium channels as voltage-gated calcium channel (VGCC) or transient receptor potential calcium channel (TRP). It could be noted that α_1_-AR are also known to be coupled to β-arrestin (that activates mitogen-activated protein kinase pathways and that allows receptor internalization), and to the small GTP binding protein Rho A that mediates calcium sensitization. Alpha_1_-adrenergic receptors have also been shown to induce arachidonic acid in a PLC-independent manner but probably through phospholipase A2 (PLA2) activation [16,17]. These different pathways converge to mitogenic response to catecholamines and to the release of calcium. The latter effect induces the contraction of smooth muscles in physiological conditions and participates in various physiological effects, such as mydriasis, increase of blood pressure and promotion of bladder continence, for example. The use of α_1_-AR antagonists disturbs intracellular calcium flux and induces a relaxation of smooth muscles; this is why these drugs are prescribed for benign prostatic hyperplasia (BPH) [19]. 

BPH is a non-cancerous proliferation of the prostate glandular epithelium, connective tissue and smooth muscle. BPH is a common disorder affecting 50% of men over 50 years old and reducing significantly their quality of life [20]. The widely held concept is that BPH leads to prostate enlargement that impinges upon the prostatic urethra and bladder outlet which is responsible of mechanical obstruction to urinary outflow and bladder detrusor muscle irritability. Moreover, the increase of smooth muscle tone at the prostate and the bladder neck accentuates the bladder outlet obstruction. The whole of these mechanisms results to lower urinary tract symptoms (LUTS), such as storage symptoms (urgency, frequency and nocturia) and voiding symptoms (weak stream, urinary retention and hesitancy) [19,20,21,22]. However, it is noteworthy that other studies questioned the causal relationship between prostatic enlargement, bladder outlet obstruction and LUTS in men with clinical BPH revealing that the mechanism by which BPH causes LUTS needs to be further investigated [23].

The treatment of BPH and associated LUTS can be achieved using three major therapeutic strategies. Firstly, prostate development is controlled by the testosterone derivate dihydrotestosterone (DHT). Thereby, the use of the 5-α reductase inhibitors (5-ARI), such as Dutasteride or Finasteride, that impede the conversion of testosterone into DHT, reduces the serum and intraprostatic DHT concentration and allows the slowdown of BPH progression [24]. Secondly, the isoenzymes PDE5 (phosphodiesterase type 5), highly expressed in LUT tissues, degrade the cyclic Guanosine Monophosphate (cGMP) inducing vessels vasoconstriction. Hence, PDE5 inhibitors, such as Tadalafil, increase the cGMP level resulting in vasodilation through activation of the endothelial Nitric Oxide Synthase–Nitric Oxide–cGMP pathway, thereby allowing relaxation of LUT tissues [25,26]. At last, the use of α_1_-AR antagonists (also called α-blockers) turned out to be very effective. Three α_1_-AR subtypes are found in LUT tissues. The α_1A_-AR subtype is predominant and is located in urethra, bladder neck and in the prostate stroma and smooth muscle (although both mRNA of α_1A_- and α_1D_-AR were found in this tissue) and it mediates prostate contractility [27,28,29]. The α_1B_-AR subtype is expressed by vascular tissue and is less abundant than α_1A_- and α_1D_-AR in male ureters [30]. Finally, α_1D_-AR subtype is also importantly expressed. It is found in bladder and sacral region of the spinal cord [27,29] and it was demonstrated that bladder smooth muscle tissue obtained from surgical patients express predominantly the α_1D_-AR subtype at mRNA level, a result that was confirmed at a protein level by competition analysis assay [31]. Moreover α_1D_-AR subtype was described to be increased in expression and function in models of bladder hypertrophy [32]. This receptor subtype takes part in bladder contraction. 

The selectivity to α_1A_- and α_1D_-AR antagonists presents several advantages. It allows these antagonists to better target prostate and bladder tissues without displaying side-effects, such as blood pressure disturbance driven by the α_1B_-AR subtype. Moreover, even if α_1A-_AR antagonists are very effective in relaxing prostate smooth muscle, their combination with α_1D_-AR antagonists is more effective to improve LUT symptom scores in men with BPH because α_1D_-AR antagonists also relieve bladder symptoms [33]. Finally, it was shown that the tissues of BPH display decreased expression of α_1B_-AR and increased expression of α_1A_- and α_1D_-AR subtypes mRNA, compared to normal prostatic tissue, leading to the suggestion that both α_1A_- and α_1D_-AR contribute to BPH development [34,35,36,37].

Combination disposals using an α-blocker and 5-ARI or PDE-5 inhibitor, anticholinergic agents or β-3-agonists can also be prescribed for the treatment of BPH [20,21,38].

Seven α_1_-AR antagonists are used for the treatment of BPH and associated LUTS. Silodosin is marketed worldwide and prazosin, alfuzosin, doxazosin, terazosin and tamsulosin are used in most western countries. Naftopidil is used for the same indication but only in Japan. Each α_1_-AR antagonist possesses a different selectivity for each α_1_-AR subtype. Alfuzosin, doxazosin, prazosin and terazosin block the three α_1_-AR subtypes, they are called non-selective α_1_-AR antagonists [16,17,27]. Conversely, silodosin is α_1A_-AR-selective antagonist and tamsulosin and naftopidil are more selective for α_1A_ and α_1D_ than for α_1B_ [27]. Concerning naftopidil, the studied carried out by Michel et al., did not allow to observe any selectivity for naftopidil in cloned α_1_-AR subtypes transiently expressed in COS cells [39]. On the contrary, naftopidil was described to bind more specifically α_1A_- and α_1D_-AR [40]. In order to precise their results, experiments using membrane preparations from CHO cells stably expressing the cloned human *α_1_-AR* genes showed that naftopidil has 17- and 3-fold higher potency for α_1D_-AR than for the α_1B_- and α_1A_-AR, respectively [41]. Yuan’s team showed similar results through docking studies and on rat functional assay in vitro and highlighted that naftopidil used as a racemate, as well as its S- and R- enantiomers had similar blocking activity on α_1_-AR subtypes [42,43]. However, a recent work contradicted these previous observations and showed that naftopidil affinity is α_1A_ > α_1B_ > α_1D_ [44]. These discrepancies are confusing but variations on affinities are frequently observed between laboratories and it could be suggested that global view of data of different laboratories is required to precise ligand binding affinity.

Naftopidil, named Flivas™, has been marketed in Japan for BPH and associated LUTS treatment by Asahi Kasei Corporation since 1999 and several clinical trials and prospective studies demonstrated that naftopidil appears efficient for the treatment of BPH and LUTS without major adverse effects [36,45]. Naftopidil which displays selectivity for α_1D_-AR expressed in the bladder, was reported to improve storage symptoms compared to tamsulosin [46,47,48]. Some studies did not find significant difference in IPPS (International Prostate Symptom Score) and quality of life between these two α_1_-AR antagonists [49], whereas other studies showed that naftopidil also increased quality of life parameter [50]. To have a global vision on naftopidil effect on LUTS associated with BPH, a meta-analysis was carried out by the Cochrane library [22]. This analysis included 22 studies with randomised 2223 participants and compared the efficacy of naftopidil to that of tamsulosin and silodosin on several outcomes. The conclusion revealed that compared to tamsulosin, naftopidil had similar effect on urological symptoms score, quality of life and adverse events. The same conclusion was obtained when naftopidil was compared with silodosin, but a substantial reduction of sexual adverse events was observed with naftopidil [22]. Thus, naftopidil seems to be as potent and tolerable as the other α_1_-AR antagonists. However, as studies evaluated naftopidil effects only on Asian men, it certainly led to bias and it would be required to test its effect on other populations.

In clinical practice, the optimal dose of naftopidil is ranging between 25 and 75 mg/day for Japanese men [45]. It has been demonstrated that, after oral administration of naftopidil, 80–95% of the dose is rapidly absorbed, widely distributed and its half-life not exceeds 3h [51]. After a single dose of 50mg, the maximal plasma concentration of naftopidil (plus its metabolites) is in the range of 300–600 nM [52]. Naftopidil is clinically used as a racemate. Its bioavailability in humans only reaches 18%, suggesting an important first-pass metabolism [52]. In this context, Zhu and colleagues showed that the hepatic metabolism associated isoenzymes CYP2C9 and CYP2C19 are involved in naftopidil metabolism, mainly by its demethylation and hydroxylation [53]. In addition, plasma levels and half-life times of naftopidil after oral administration are increased in patients with hepatic dysfunction [51], comforting the importance of hepatic metabolism in pharmacokinetic properties of naftopidil. A study carried out in rats confirmed this fist-bypass metabolism and showed that naftopidil S-enantiomere bioavailability is higher than racemate and twice higher than R-enantiomer after oral administration [54]. However, R-enantiomere was more widely distributed in peripheral tissues with high concentrations found in prostate, suggesting stereoselective pharmacokinetic [54]. Buccal films of naftopidil allowing its intra-oral administration are currently undergoing development and evaluation in order to overcome its hepatic by-pass and thereby to enhance its bioavailability [55]. Finally, this compound is well tolerated at up to 100 times the pharmacologically active dose and its therapeutic index is in the range of 4.4–6.7, supporting that naftopidil displays a broad therapeutic range [52].

In parallel of studies showing its activity for BPH treatment, several results demonstrated that naftopidil exhibited certain anti-cancer properties in vitro, in vivo as well as in clinic.

## 3. Anti-Cancerous Properties of Naftopidil

### 3.1. Cytostatic Effects of Naftopidil In Vitro

Several studies showed that naftopidil can exert anti-proliferative effects on cancer cell lines (Table 1).

In this context, Kanda et al., studied the effect of naftopidil on growth of human androgen sensitive, androgen receptor-positive LNCaP cell line and androgen insensitive, androgen receptor-negative PC-3 cell line [62]. They showed that naftopidil had an anti-proliferative effect on both cell lines with IC50 around 20 and 30 µM respectively and induced a cell cycle arrest with a blockade in G0/G1 phase. This anti-proliferative effect was also observed in androgen low-sensitive, androgen receptor-positive E9 cell line (that derives from LNCaP) [56] implying that the antiproliferative effect of naftopidil is not related with androgen sensitivity of the cells. Molecular mechanism analysis showed that in androgen sensitive cell line LNCaP, p27^kip1^ and p21^cip1^ were strongly up-regulated, whereas only the former was increased in E9 cell lines and only the latter was increased in PC-3 androgen insensitive ones. This result implies that the antiproliferative effect of naftopidil depends on cellular context. Moreover, whereas naftopidil had no effect on Akt activity in androgen-sensitive cell line (LNCaP), it inhibited Akt phosphorylation on ser^473^ in androgen-insensitive cell line, PC-3. Authors suggested that this result could account for p21^cip1^ inhibition in PC-3. Finally, naftopidil did not modulate p53 expression in both cell lines, ruling out p53 implication in naftopidil-induced p27^kip1^ and p21^cip1^ proteins [62]. Naftopidil was also demonstrated to have an anti-proliferative effect on the renal cancer cells lines ACHN an Caki-2 in the same range of concentrations. In fact, it reduced cell proliferation that was accompanied by an arrest in the G0/G1 phase of the cell cycle, a decrease of Cyclin dependent kinase-2 (Cdk-2) expression (which is required for the transition of cell cycle phases) and increase of p21^cip1^ [57]. As well, naftopidil was proven to reduce proliferation of colon adenocarcinoma cells HT29 [60] and that of ovarian cancer cell lines SKOV3 and IGROV1-R10 in a dose-dependent manner [61]. Even if this anti-proliferation effect was accompanied by an increase of p21^cip1^ and p27^kip1^ in SKOV3 cell line, the expression of these proteins was not disturbed in IGROV1-R10 cell lines supporting that naftopidil anti-proliferative effect was cell-context dependent (personal data). This antiproliferative effect was also observed in cells in the microenvironment of the cancer cells. For example, in Hori’s study, naftopidil also reduced the growth of the fibroblasts cells PrSC derived from the prostate stroma as well as their secretion of IL-6, a growth factor for most prostate cancer cells, suggesting that naftopidil could interfere with tumour-stroma interactions [56]. This drug also increased p21 expression in HUVEC cells preventing their proliferation in vitro and giving to naftopidil an anti-angiogenic property [57].

### 3.2. Cytotoxic Effects of Naftopidil In Vitro

Naftopidil was also described to exert cytotoxic effects in several cancer cell lines when used as a single agent (Table 1). Actually, naftopidil reduced cell viability in different models such as bladder and renal cancer cells [58,59]. Moreover, it was shown to induce apoptosis of mesothelioma cells NCI-H28, NCI-H2052, NCI-2452 and MSTO-211H by activating caspase 3 and 8 [63,64] and increasing TNF-α mRNA expression and Fas-Ligand secretion in NCI-H2052 however, this effect is only observed for high concentrations. Several results also demonstrated that this α_1_-AR antagonist also triggered prostate cancer, cervical and gastric cancer cells apoptosis [65,66].

In most models, naftopidil appeared cytotoxic in in vitro assays at concentrations higher than 50 µM [62,63,64,66]. Under this threshold, naftopidil exerted a cytotoxic activity when it was used in combination with other anti-neoplasic strategies. In fact, naftopidil displayed an additive cytotoxic effect with radiotherapy in PC-3 cells [67]. Moreover, by an indeterminate mechanism, naftopidil induced apoptosis of LNCaP and PC-3 prostate cancer cells in combination with docetaxel, but not that of prostate stromal cells PrSC [68]. As for ovarian cancer cells SKOV3 and IGROV1-R10, whereas naftopidil only exerted a cytostatic effect when used as a single agent, it transcriptionally up-regulated Bim, Puma and Noxa pro-apoptotic protein expression. The combination of naftopidil with the BH3-mimetic targeting Bcl-x_L_ ABT-737 or the MEK inhibitor Trametinib increased the [pro]/[anti-apoptotic] ratio in favour of the pro-apoptotic proteins, leading these cancer cell lines and high-grade ovarian cancer Patient-Derived Organoids (PDO) to apoptosis [61]. It is noteworthy that in most studies, the anti-cancer effects of naftopidil were not assessed in non-malignant cell, a control condition that would permit to evaluate therapeutic margins. Its effect was however evaluated on T1074 cell (non-malignant ovarian epithelial cells). Naftopidil at 50 µM has an anti-proliferative effect; however, its combination with Trametinib did not trigger apoptosis as observed in malignant SKOV3 and IGROV1-R10 cell lines, suggesting that naftopidil only sensitises ovarian malignant cell to targeting therapies [61].

### 3.3. Anti-Cancerous Effects of Naftopidil In Vivo

To evaluate its efficacy in vivo, naftopidil anti-cancerous effects were analysed in xenografted mice models (Table 1). Studies highlighted that naftopidil is a well-tolerated molecule as it did not provoke weight reduction in the treated mice [59,63]. Moreover, naftopidil was proven to be an efficient cytotoxic drug in several cancer types as it reduced the volume of tumours [56,62]. This effect was accompanied by a decrease in Ki-67 index [56], and also an increase in p21 staining [62]. Naftopidil exhibited cytotoxic effects in xenografts of mesothelioma, renal carcinoma and bladder cancer cells [57,59,63]. Naftopidil also reduced PC-3 xenograft tumour growth alone and more drastically in combination with radiotherapy [67] or docetaxel [68], such as in in vitro experiments. Finally, naftopidil exerted anti-angiogenic properties through reduction of the microvessels density (MVD) in renal carcinoma cell line (ACHN) and prostate cancer cell (PC-3) xenograft models, but also, in-patient renal carcinoma xenografted into nude mice [57,62].

All these studies were carried out at the dose of 10 mg/kg/day naftopidil, or twice a week for Mikami’s study [63]. This suggests that this dose is tolerable and effective in the model of cancer tested. Moreover Kanda et al., showed that increasing the dose to 100 mg/kg/day did not improve naftopidil efficacy [62]. Taken together, these observations suggest that naftopidil exerts anti-cancerous properties in vitro and in vivo in several cancer types.

### 3.4. Clinical Evidence of Naftopidil Anti-Cancer Effects

The anti-cancer effect of naftopidil in clinic was observed thanks to a retrospective study that was carried out in Memorial Hospital in Tokyo [65]. In this study, prostate cancer incidence has been evaluated in patients who have received either naftopidil (*n* = 766) or tamsolusin (*n* = 1015) between 2003 and 2010. As expected, prostate cancer incidence was correlated with the level of PSA concentration. Surprisingly, this incidence was lower in the naftopidil group than in tamsulosin group from 3 months of treatment (1.8% versus 3.1% OR = 0.46 *p* = 0.035) and this difference is accentuated with the duration of treatment 0.46 (*p* = 0.081) for 12 months and 0.16 (*p* = 0.039) for 36 months. Moreover, these authors also showed that prostate cancer cells from men treated with naftopidil over-expressed p21 and under-expressed Bcl-2 compared with men exposed to tamsulosin or no treatment, suggesting that naftopidil acted through inhibition of cell cycle progression and perturbation of apoptosis member expression in clinic. These encouraging results allowed naftopidil to be considered as a promising candidate for prostate cancer management in chemoprevention although it should be kept in mind that retrospective cohort studies, even if results can be easily generalizable to real-world situation, are subjected to bias because of absence of randomization. Thus, randomized controlled trials are required to evaluate causal relationship between naftopidil treatment and reduction of prostate cancer incidence [69,70].

## 4. Molecular Mechanisms Involved in the Anti-Cancerous Properties of Naftopidil

### 4.1. Involvement of the Chemical Structure

The chemical structure of α_1_-AR antagonists seems to play a major role in the capacity of these molecules to display anti-cancer properties and Kyprianou and colleagues suggested that their anti-cancer activity is quinazoline/piperazine-dependent. Actually, it has been demonstrated that exposure to the quinazoline-based α_1_-AR antagonists’ doxazosin, prazosin and terazosin induced apoptosis, anoikis, decreased cell growth in bladder and in several prostate cancer cell lines in vitro as well as in vivo, exerted anti-angiogenic properties and sensitised human cervical carcinoma cell lines to chemotherapy through inhibition of MDR-1 mediated drug efflux. These results have been comprehensively collected by Batty et al., [71]. On the contrary, tamsulosin, a sulphonamide-based α_1_-AR antagonist that does not possess a quinazoline or piperazine group displayed no anti-cancer property [72,73]. Interestingly, a significant decrease in the incidence of prostate cancer was also observed in clinic for the quinazoline-based α_1_-AR antagonists. Indeed, the result of a retrospective cohort study showed that men treated with doxazosin and terazosin have a 1.46 times lower relative risk to develop prostate cancer compared with non-treated men [74]. Although prospective clinical studies are lacking to support these arguments, these results encourage further studies.

**Figure 1:**
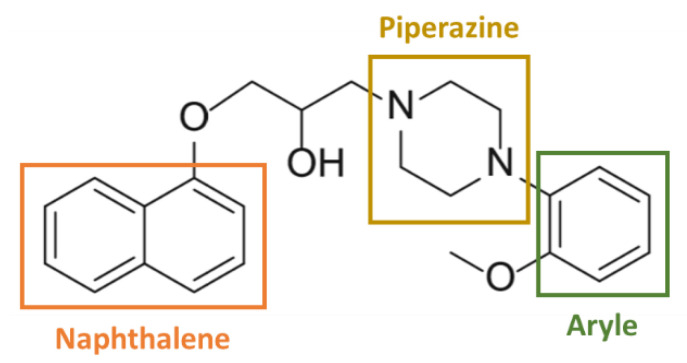
Chemical structure of naftopidil (according to [65,71,75]). Naftopidil is an aryl-piperazine based α1-AR antagonist possessing a naphthalene group.

Naftopidil is an aryl-piperazine based α_1_-AR antagonist possessing a naphthalene group (Figure 1) and its anti-cancerous properties could be dependent of these chemical groups.

Piperazine is a core scaffold for synthesis of a plethora of bioactive molecules and piperazine-based drugs possess various pharmacological activities such as anti-fungal, anti-viral, anti-depressant properties [76,77,78] and also exert anti-cancerous properties. For example, an 1-(2-aryl-2-adamantyl)piperazine derivate reduced viability of cervical, breast, pancreatic and lung cancer cells in a dose-dependent manner without exerting any toxicity on normal cell lines [79] while others seem to have HDAC (Histone deacetylase) inhibitor properties [80]. Moreover, several aryl-piperazine derivatives containing the saccharin moiety were shown to reduce cell viability of cancer prostate models in a dose-dependent manner [81]. These structure-activity relationships open the perspective of their use as anti-cancer drugs as illustrated by the anti-cancer effects of naftopidil in vitro and in vivo but also led to synthesis of naftopidil derivatives as HUHS1015 and compound **12** to improve its efficacy as it is discussed thereafter.

### 4.2. Alpha_1_-AR Independent Anti-Cancerous Action

As α_1_-AR antagonists, naftopidil and its derivatives anti-cancer actions were supposed to involve α_1_-AR pathway. In this context, it has been shown that the endogenous α_1_-AR agonist noradrenaline protected prostate cancer cells against the anti-proliferative effect of the naftopidil derivate, compound **12**, suggesting that this naftopidil analogue acted through α_1_-AR binding to exert its anti-cancerous activity [82]. It should be remarked that even noradrenaline has a high affinity for α_1_-AR, this catecholamine can also bind other adrenoreceptors as α_2_- or β-AR to exert its proliferative activity. So, the noradrenaline protective effect observed does not necessarily attest that compound **12** acts through α_1_-AR and other pathways could not be excluded. As for naftopidil, several studies described that its anti-proliferative and cytotoxic effects involved α_1_-AR independent mechanisms. In fact, naftopidil reduced mesothelioma cell viability, while the α_1D_-AR knock-down enhanced it [64]. Moreover, the α_1D_-AR stimulation led to Protein Kinase A (PKA) and PKC activation, thereby their inhibition should enhance naftopidil effects. However, the PKC inhibitor GF109203X attenuated naftopidil-induced apoptosis of mesothelioma cells [64]. Moreover, neither the PKA inhibitor H89 nor the GF109203X increased naftopidil cytoxic effects on bladder cancer cells. Furthermore, the α_1_-AR agonists’ methoxamine and phenylephrine should counteract the effects of naftopidil, which was not observed in bladder cancer models [59]. Similarly, neither the cytostatic effect of naftopidil nor its capacity to induce BH3-only protein expression in ovarian cancer cell lines were counteracted by methoxamine. Moreover, these effects were not mimicked by the other α_1A_/α_1D_-AR-selective drug BMY-7378 that also displays a phenylpiperazine moiety [61]. Lastly, naftopidil reduced proliferation of the prostate cancer cells AIDL (Androgen-Independent LNCaP), although this cell line does not express the α_1D_-AR subtype [56].

Interestingly, it has also been described that the α_1_-AR antagonists’ doxazosin and terazosin induced apoptosis of prostate cancer cells independently of α_1_-AR. In fact, these drugs induced apoptosis of PC-3 cells that was not abrogated by phenoxybenzamine, an irreversible α_1_-adrenoreceptor antagonist [72]. Moreover, these compounds induced apoptosis in DU-145 cells line that lacks α_1_-AR [73]. This suggests that naftopidil is not the only α_1_-AR antagonist exerting anti-cancerous properties independently of α_1_-AR. Finally, it should be remarked that, in all of these studies, concentrations required for anti-cancerous activities are higher than those required for α_1_-AR inhibition [34,35,36]. This finding is also an argument to support that the anti-cancerous properties of α_1_-AR antagonists probably do not involve α_1_-AR pathway.

### 4.3. Other Pathways Involved

Naftopidil was found to modulate the activity of different signaling pathways. Firstly, naftopidil reduced Akt phosphorylation in prostate and gastric cancer cells [62,66,68]. However, this effect was not observed in ovarian cancer cell lines [61].

Moreover, this α_1_-AR antagonist was described to reduce activity of the TGF-β pathway by decreasing Smad2 phosphorylation in HeLa cells but the entire molecular pathways is not described [65].

Depending on cellular context in ovarian cancer cell lines, naftopidil induced either ER stress-activated ATF4 transcription factor or JNK/c-Jun phosphorylation; both pathways leading to BH3-only protein up-regulation. Interestingly, these molecular transduction pathways are known to be activated by destabilization of microtubules [83,84] and naftopidil was described to disturb microtubules polymerization [60]. In fact, in this study, Ishii and colleagues showed that naftopidil, as well as other phenylpiperazine derivates RS100329, BMY-7378, and KN-62, were able to bind tubulin and to inhibit its polymerization. In contrast, the quinazoline-based α_1_-AR antagonist doxazosin increased the tubulin polymerization and tamsulosin or silodosin, which are respectively carboxamide- or sulphonamide-based α_1_-AR blockers, did not show any effect. Thereby, the ability to bind tubulin appears to be a specific feature of piperazine-based drugs [60]. This property was supported by the fact that AK301, a piperazine-based compound, was also known to inhibit tubulin polymerization and to induce a blockade of colon cancer cells in the G2/M phase of the cell cycle which restored their apoptosis in presence of TNF-α [85]. Microtubule-targeting agents are known to modulate the activity of several molecular pathways by disturbing microtubule dynamic and thereby exert anti-cancerous properties [83]. It could then be hypothesized that through interaction with tubulin, naftopidil could activate ER stress and JNK/c-Jun pathways, increasing pro-apoptotic protein expression and allowing naftopidil to sensitise ovarian cancer cells to pro-apoptotic strategies [61]. However, the ability of the naftopidil analogues sharing aryl-piperazine moiety to bind tubulin has not been demonstrated yet and further studies are needed.

Moreover, an in-silico drug repositioning-approach carried out to predict drugs that can modulate transcription factor activity identified naftopidil among more than 6700 drugs as a potential activator of p53 [86]. This pathway could besides be involved in cell cycle arrest and apoptosis [87] which could explain the anti-cancerous properties of naftopidil on several models, even if p53 implication was ruled out in naftopidil-induced BH3- only increase in ovarian cancer cells [61].

Taken together, these observations strongly suggest that naftopidil acts as an anti-cancer agent independently of its α_1_-AR antagonist role. However, the molecular targets of AR-independent effects remain unknown and certainly depend on cellular context.

### 4.4. Anti-Cancerous Properties of Naftopidil Analogues

Interestingly, the naftopidil major metabolite HUHS190, also reduced cell viability of prostate, bladder and renal cancer cell lines in dose-dependent manner [88]. In this context, and in order to obtain more effective anti-cancer drugs, different naftopidil analogues were synthesized. Thus, naftopidil derivates containing methyl phenylacetate moiety exhibited α_1_-AR antagonist properties but their potential anti-cancerous activity has not been studied yet [89]. Other naftopidil-based aryl-piperazine analogues were developed and among them, some exert anti-cancerous properties against prostate cancer cells, such as derivates containing a bromophenol moiety [75,90].

**Figure 2:**
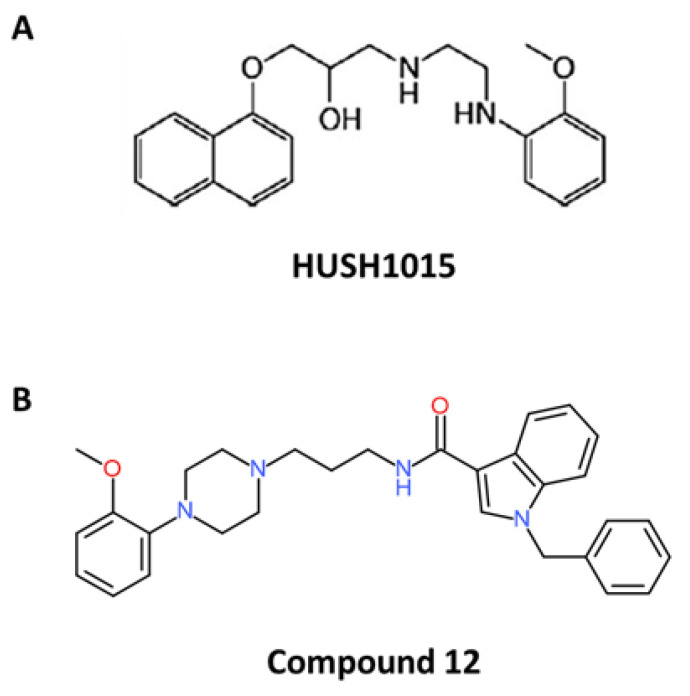
Chemical structures of naftopidil analogues (according to [82,91]). (**A**) Chemical structure of HUHS1015 and (**B**) chemical structure of compound **12**.

Among all naftopidil analogues, the lead molecule HUHS1015 was widely studied such as another drug called “compound **12**” (Figure 2) (Table 2).

### 4.5. HUHS1015

Nishizaki and colleagues synthesized 21 naftopidil analogues and among them HUHS1015 seemed to present interesting anti-cancerous properties [92].

In vitro, HUHS1015 reduced mesothelioma cell lines proliferation by inducing an arrest in G0/G1 phase of the cell cycle at 10 µM and induced their necrosis and apoptosis at 15 µM after a 24 h treatment [91]. Moreover, HUHS1015 was able to reduce cell viability in other several models, notably it induced cell death in lung, liver, gastric, bladder and renal cancer cells [93]. Concerning mesothelioma cell lines, it is interesting to note that HUHS1015 is less cytotoxic for the non-malignant MeT-5A cell line than for malignant ones.

Molecular pathways involved in mesothelioma cell lines showed that caspase 3 and 4 are activated, but not caspase 8 and 9, suggesting that HUHS1015 induced apoptosis not through canonical extrinsic and intrinsic pathways but perhaps through reticulum stress (known to activate apoptosis through caspase 4) or through independent-caspase pathway [93].

In addition, a 24h-treament with 15 µM HUHS1015 induced both necrosis and apoptosis in cisplatin-resistant MKN28 and MKN45 gastric cancer cells [94]. Interestingly, HUHS1015 triggered MKN45 cells apoptosis by activating caspase 3, 4 and 8 while it acted by a caspase-independent way, probably through nuclear accumulation of AMID (Apoptosis-inducing factor-homologous Mitochondrion-associated Inducer of Death) in MKN28 cell line [95].

HUHS1015 was also able to bring colorectal cancer cell lines Caco-2 and CW2 in apoptosis by inducing mitochondria damages [96].

Moreover, HUHS1015 modulated the expression of proteins implicated in apoptosis regulation, which could explain its ability to induce this type of cell death. Indeed, in mesothelioma cells, 15 µM HUHS1015 increased the mRNA expression of Puma, Hrk and Noxa, implicated in the intrinsic apoptosis pathway control [91]. It also increased the mRNA expression of Bax and Bad in colorectal cancer cells [96], such as naftopidil was able to transcriptionally induce BH3-only proteins expression in ovarian cancer cells [61]. Like naftopidil in mesothelioma cells, HUHS1015 also increased expression of factors implicated in the extrinsic apoptosis pathway, such as the cytokine TNF-α in MKN45 gastric cells; however, this effect has been called into question due to the very high concentrations of HUHS1015 used [63,94].

In vivo, HUHS1015 was described to be well tolerated by mice, as it did not induce a loss of weight and it reduced the tumour volume in mesothelioma, gastric and colorectal cancer cells xenografted mice [91,94,96]. Moreover, HUHS1015 seemed to be more efficient than naftopidil on gastric and colorectal cancer models as it induced a stronger decrease of tumour for the same dose used [94,96].

### 4.6. Compound **12**

Huang and colleagues designed eleven compounds, compound **2** to compound **12**, derived from naftopidil [82]. For all these derivates, the 2-hydroxypropane of naftopidil was replaced by an amide structure in order to improve their α_1A/1D_ binding affinity and an indole substituent was introduced because it was suggested to enhance their anti-cancerous properties. Among these novel synthesised molecules, compound **12** possessed a large benzyl group at the indole-N-position that increased its flexibility and was suggested to strengthen its affinity to α_1A/1D_-AR, lower its binding to α_1B_-AR through its hydrophobic properties and favour anti-cancerous properties of α_1_-AR antagonists [82,97]. Compound **12** showed the highest cytotoxic activity against the PC-3, DU145, and LNCaP prostate cancer cell lines in vitro and induced an arrest in the G0/G1 phase of the cell cycle, such as naftopidil and HUHS1015 [82]. Beyond 20 µM, compound **12** induced apoptosis of prostate cancer cells, as observed by activated caspase 3 and phosphatidylserine exposure. As HUHS1015, compound **12** was able to modulate Bcl-2 family member expression by inducing Bax and Bcl-2 mRNA expression [82]. To our knowledge, this compound was not used in in vivo experiments.

Taken together, naftopidil anti-cancerous properties allowed the development of several analogues. Among them, HUHS1015 and compound **12** display anti-cancerous activities in several cancer types at lower concentrations than naftopidil, suggesting their better efficacy. However, other studies are required to decipher molecular pathways involved and to evaluate the clinical safety of these two analogues.

## 5. Discussion

Collectively, naftopidil shows anti-cancer properties on several cancer models. In vitro, naftopidil has an anti-proliferative effect, presumably by modifying the expression of proteins regulating cell cycle progression [56,57,60,61,62]. As it can inhibit cancer cell growth when used as a single agent, naftopidil could be regarded as a cytostatic drug which could slow proliferation and generate a lap of disease stabilisation that will delay the introduction of cytotoxic drugs and improve time of progression, quality of life and survival. Yamada’s study suggested that naftopidil reduced prostate cancer incidence and that this compound could be seen as a potential chemopreventive treatment. However, its antiproliferative action could also allow this compound to be suggested as a possible maintenance treatment for spacing out recurrence episodes. Other clinical studies are required to evaluate this point.

Naftopidil can also exert cytotoxic effects when used as a single agent [58,59,62,63,64,65,66], but in other models it was also able to induce apoptosis only when combined with other antineoplasic drugs in vitro, in vivo as well as ex vivo in high grade serous ovarian cancer PDO models [61,67,68]. This result suggests that naftopidil could be regarded as a possible chemotherapeutic adjuvant due to its capacity to sensitise cancer cells to other therapies. A combination with other molecules seems to be an interesting strategy as it could target different pathways that act synergistically. This could allow reduction of dose and thereby limitation of possible side effects. Studies deciphering molecular pathways activated by naftopidil would also help to find new relevant therapeutic combinations to empower naftopidil efficacy. However, these studies are lacking and efforts have to be made to fill the gap.

Interestingly, anti-cancerous effects of naftopidil seem to be independent of its α_1_-AR antagonist property [56,59,61,64] and, depending on cellular context, naftopidil is able to modulate activity of various signaling pathways [60,61,62,65,86].

The anti-cancerous properties of naftopidil has promoted the development of numerous derivatives to improve α_1_-AR affinity for prostate targeting or cytostatic/cytotoxic activity [75,82,90,91,92,93,94,95,96]. Indeed, naftopidil cytostatic and cytotoxic properties are reached at high concentrations in vitro and reducing effective concentrations through more effective compounds would facilitate is use in clinic. It is interesting to note that Colciago et al., synthesised a compound derived from WB4101: A175, that binds with a strong affinity α_1D_-AR [98]. They demonstrated that this compound had an anti-proliferative effect in androgen-insensitive prostate cancer cells PC3 that strongly expressed α_1D_-AR at the mRNA level but had no effect on DU145 cells that did not express this receptor. This study showed that mRNA expression of α_1A_-AR was highly expressed in less aggressive and androgen-sensitive prostate cancer cells suggesting that α_1A_-AR antagonists might be more useful to counteract cell proliferation in the first steps of cancer. This is in agreement with Thebault’s work showing that LNCaP cells expressed α_1A_-AR and that the inhibition of these receptors prevented cancer epithelial cell proliferation [99]. Conversely, α_1D_-AR was expressed in androgen-independent prostate cancer cells and α_1D_-AR antagonists could be more useful in this type of cancer. So, the improvement of α_1_-AR selectivity (as made for compound **12**) could be important in prostate cancer management, especially because it was suggested that α_1D_-AR is overexpressed in many untreated patients with advanced prostate cancer and thus could be regarded as an interesting target [100]. For other types of cancer, it would be interesting to improve the anti-proliferative effects of the analogues by disrupting the dynamics of tubulin (as arylpiperazine compounds do) or by blocking the cell cycle in the G0/G1 phase. These types of compounds have been shown to be good adjuvants for sensitizing to conventional chemotherapies and allowing for the spacing of treatment cycles. In addition, it would prevent the acquisition of mutations and the development of chemoresistance.

Although there is evidence suggesting that naftopidil may be considered a potential candidate for drug repurposing, some questions arise. Naftopidil is approved for BPH/LUTS treatment in Japan, but not in other countries, because of the lack of non-Asian randomized clinical trials and placebo-controlled trials [22]. As the Asian population displays a metabolic phenotype different to that of the Caucasian or African population, one could then argue that naftopidil would not be metabolized in the same way, which could lead to side-effects or lack of efficacy. Moreover, naftopidil is used for BPH/LUTS treatment in men and it has never been used on women; clinical trials on women are then needed to prove is safety without major adverse effects. Finally, naftopidil safety was not evaluated beyond 18 weeks of treatment in clinical trials [101]. As its possible use in cancer management would need longer courses of treatment, clinical trials are required to ensure its safety.

Taken together, naftopidil displays cytostatic and cytotoxic properties in several in vitro and in vivo models. Its capacity to slow cancer cell proliferation, the result from Yamada’s study and its good tolerability could enable naftopidil to be considered as a potential candidate for cancer prevention or maintenance treatment. Moreover, it could also be considered as a good adjuvant due to its capacity to potentialize anti-cancer therapies. However, its molecular targets need to be more deeply investigated to relevantly sensitise cancer cells to other anti-tumoural drugs. Finally, its anti-tumoural efficacy has to be confirmed and evaluated in large cohort prospective clinical studies. Naftopidil still has a long way to clinically prove its efficacy and tolerability in cancer management, but the encouraging results make naftopidil an interesting candidate to drug repurposing and incite to pursuit investigations on its anti-cancerous properties.

## Figures and Tables

**Table 1:** Anti-cancerous properties of naftopidil.

Cancer Models					
Organs	Study	Models	Drug Effects	Observations	Authors
Bladder	in vitro	253J, 5637, KK-47, T24 and UM-UC-3	Cell viability reduction	-	Gotoh et al., 2012 [56]
	in vitro	KK-47, 5637, T-24	Cell viability reduction	-	Nakagawa et al., 2016 [57]
	in vivo	KK-47	Tumour volume reduction	-	Nakagawa et al., 2016 [57]
Cervival	in vitro	HeLa	Cell viability reduction by induction of apoptosis	Naftopidil supresses phosphorylation of Smad-2 induced by TGF-β	Yamada et al., 2013 [58]
Colon	in vitro	HT29	Cell viability reduction	-	Ishii et al., 2015 [55]
Gastric	in vitro	HGC27	Cell viability reduction by induction of apoptosis	Naftopidil reduces Akt phosphorylation	Nakamura et al., 2018 [59]
Mesothelioma	in vitro	NCI-H28, NCI-H2052, NCI-H2452, MSTO-211H	Cell viability reduction by induction of apoptosis	-	Masachika et al., 2013 [60]
		NCI-H2052	Cell viability reduction by induction of apoptosis	Naftopidil increases TNF-α mRNA expression and Fas-L secretion	Mikami et al., 2014 [61]
	in vivo	NCI-H2052	Tumour volume reduction	-	Mikami et al., 2014 [61]
Ovarian	in vitro	IGROV1-R10 and SKOV3	Anti-proliferative effect alone and apoptosis induction in combination with ABT-737 or Trametinib	Naftopidil increases Bim, Puma and Noxa protien expression	Florent et al., 2020 [62]
Prostate	in vitro	LNCaP, E9 and PrSC	Anti-proliferative effect (blockade in G0/G1 phase of the cell cycle)	Naftopidil increases p21 and p27 protein expression and reduces IL-6 secretion	Hori et al., 2011 [53]
		LNCaP and PC-3	Anti-proliferative effect (blockade in G0/G1 phase of the cell cycle)	Naftopidil increases p21 and p27 protein expression and reduces Akt phosphorylation	Kanda et al., 2008 [52]
		DU145, LNCaP and PC-3	Cell viability reduction	-	Gotoh et al., 2012 [56]
		LNCaP	Cell viability reduction by induction of apoptosis	-	Yamada et al., 2013 [58]
		LNCaP and PC-3	Anti-proliferative effect alone and apoptosis induction in combination with Docetaxel	-	Ishii, 2018 [63]
		PC-3	Cell viability reduction enhanced with radiotherapy	Naftopidil reduces Akt phosphorylation and suppresses radiotherapy-induced MnSOD	Iwamoto et al., 2017 [64]
	in vivo	E9 and PrSC	Tumour weight reduction	Naftopidil reduces Ki-67 staining and MVD	Hori et al., 2011 [53]
		PC-3	Tumour volume reduction	Naftopidil reduces Ki-67 staining and MVD and increases p21 staining	Kanda et al., 2007 [52]
		LNCaP and PrSC	Tumour volume reduction reduction enhanced with Docetaxel	Naftopidil reduces Ki-67 staining and tumour-bone interface and increases cleaved-caspase 3 staining	Ishii et al., 2018 [63]
		PC-3	Tumour volume reduction reduction enhanced with radiotherapy	Naftopidil reduces Ki-67 staining	Iwamoto et al., 2017 [64]
	Prospective study		Naftopidil exposure, for at least 3 months, is associated with a lower incidence of prostate cancer than tamsulosin	Naftopidil treatment reduces Bcl-2 and increased p21 expression in prostate cancer cells from men	Yamada et al., 2013 [58]
Renal	in vitro	ACHN and Caki-2	anti-proliferative effect (blockade in G0/G1 phase of the cell cycle)	Naftopidil increases p21 and reduces Cdk2 protein expression	Iwamoto et al., 2013 [54]
		786-O, ACHN and RCC4-VHL	cell viability reduction	-	Gotoh et al., 2012 [56]
	in vivo	ACHN	Tumour weight reduction	Naftopidil increases p21 staining and reduces Ki-67 and Cdk2 staining and MVD	Iwamoto et al., 2013 [54]
		Patient RCC	Tumour dimensions	Naftopidil reduces MVD	Iwamoto et al., 2013 [54]
**Non Cancer Models**					
Endothelial	in vivo	HUVEC	Anti-proliferative effect (blockade in G0/G1 phase of the cell cycle)	Naftopidil increases p21 protein expression	Iwamoto et al., 2013 [54]

**Table 2:** Anti-cancerous properties of naftopidil analogues HUHS1015 and compound **12.**

		Cancers		Cell Lines	Drug Effects	Observations	Authors
**Naftopidil Analogues**	**HUHS1015**	Bladder	in vitro	253J, 5637, KK-47, TCCSUP, T24 and UM-UC-3	Cell viability reduction by apoptosis induction	-	Kanno et al., 2013 [89]
		Colorectal	in vitro	Caco-2 and CW2	Cell viability reduction by inducing apoptosis and necrosis	HUHS1015 induces mitochondrial damage and increases Bad, Bax and Puma mRNA expression	Kaku et al., 2016 [92]
			in vivo	CW2	Tumour volume reduction and survival rate increase	-	Kaku et al., 2016 [92]
		Gastric	in vitro	MKN28 and MKN45	Cell viability reduction by induction of apoptosis and necrosis	HUHS1015 increases TNF-α mRNA and protein expression	Kaku et al., 2015 [90]
				MKN28 and MKN45	Cell viability reduction by apoptosis induction	-	Kanno et al., 2013 [89]
			in vivo	MKN45	Tumour volume reduction and survival rate increase	-	Kaku et al., 2015 [90]
		Liver	in vitro	HepG2 and HuH-7	Cell viability reduction by apoptosis induction	-	Kanno et al., 2013 [89]
		Lung	in vitro	A549, SBC-3 and Lu-65	Cell viability reduction by apoptosis induction	-	Kanno et al., 2013 [89]
		Mesothelioma	in vitro	MSTO-221H, NCHI-H28, NCI-H2052 and NCI-H2452	Cell viability reduction by anti-proliferative effect (blockade in G0/G1 phase of the cell cycle) and induction of apoptosis and necrosis	HUHS1015 increases Puma, Noxa, Bad and HRK mRNA expression	Kaku et al., 2014 [88]
			in vivo	NCI-H2052	tumor volume reduction	-	Kaku et al., 2014 [88]
		Prostate	in vitro	DU145, LNCaP and PC-3	Cell viability reduction by apoptosis induction	-	Kanno et al., 2013 [89]
		Renal	in vitro	ACHN, RCC4-VHL and 786-O	Cell viability reduction by apoptosis induction	-	Kanno et al., 2013 [89]
	**Compound 12**	Prostate	in vitro	PC-3, DU145, and LNCaP	Cell viability reduction by anti-proliferative effect (blockade in G0/G1 phase of the cell cycle) and induction of apoptosis	Compound **12** increases Bcl-2 and Bax mRNA expression	Huang et al., 2015 [77]

## Abbreviations

α_1_-AR	α_1_-Adrenergic Receptors
5-ARI	5-α Reductase Inhibitors
Akt	protein kinase B
AMID	Apoptosis-inducing factor-homologous Mitochondrion-associated Inducer of Death
ATF4	Activating Transcription Factor 4
AMPK	Adenosine Monophosphate-actived Protein Kinase
Bad	Bcl_2_-Associated agonist of cell death
Bax	Bcl-2–associated X
Bcl-2	B-cell lymphoma 2
Bim	Bcl-2 like 11
Bcl-x_L_	B-cell lymphoma X long isoform
BPH	Benign Prostatic Hyperplasia
Cdk-2	Cyclin dependent kinase-2
cGMP	cyclic Guanosine Monophosphate
CYP2C9	Cytochrome P450 2C9
CYP2C19	Cytochrome P450 2C19
DAG	Diacylglycerol
DHT	Dihydrotestosterone
ER	Endoplasmic Reticulum
FDA	Food and Drug Administration
HDAC	Histone Deacetylase
Hrk	Harakiri
IP3	Inositol 1,4,5-triphosphate
IP3-R	IP3-Receptor
JNK	c-Jun NH2-terminal Kinase
LUTS	Lower Urinary Tract Symptoms
Mcl-1	Myeloid cell leukemia-1
MVD	Microvessels Density
Noxa	Phorbol-12-myristate-13-acetate-induced protein 1
PARP	Poly (ADP-Ribose) Polymerase
PDE5	Phosphodiesterase type 5
PDO	Patient-Derived Organoids
PLC	phospholipase C
PI3K	Phosphoinositide 3-Kinase
PIP2	Phospholipid phosphatydylinositol-4,5-bisphosphate
PKA	Protein Kinase A
PKC	Protein Kinase C
Puma	p53 Up-regulated Modulator of Apoptosis
TGF-β	Tumour Growth Factor-β
TNF-α	Tumour Necrosis Factor-α
TRP	Transient Receptor Potential calcium channel
VGCC	Voltage-Gated Calcium Channel
